# CUEDC1 inhibits epithelial-mesenchymal transition via the TβRI/Smad signaling pathway and suppresses tumor progression in non-small cell lung cancer

**DOI:** 10.18632/aging.103329

**Published:** 2020-10-25

**Authors:** Yue Cui, Yang Song, Shi Yan, Mengru Cao, Jian Huang, Dexin Jia, Yuechao Liu, Shuai Zhang, Weina Fan, Li Cai, Chunhong Li, Ying Xing

**Affiliations:** 1 The Fourth Department of Medical Oncology, Harbin Medical University Cancer Hospital, Harbin, China; 2 Department of Orthopedic Surgery, The Second Affiliated Hospital of Harbin Medical University, Harbin, China

**Keywords:** CUEDC1, metastasis, EMT, TβRI/Smad signaling pathway, Smurf2

## Abstract

Lung cancer remains the most lethal cancer worldwide because of its high metastasis potential. Epithelial-mesenchymal transition (EMT) is known as the first step of the metastasis cascade, but the potential regulatory mechanisms of EMT have not been clearly established. In this study, we first found that low CUEDC1 expression correlated with lymph node metastasis in non-small cell lung cancer (NSCLC) patients using immunohistochemistry (IHC). CUEDC1 knockdown promoted the metastasis of NSCLC cells and EMT process and activated TβRI/Smad signaling pathway. Overexpression of CUEDC1 decreased the metastatic potential of lung cancer cells and inhibited the EMT process and inactivated TβRI/Smad signaling pathway. Immunoprecipitation (IP) assays showed that Smurf2 is a novel CUEDC1-interacting protein. Furthermore, CUEDC1 could regulate Smurf2 expression through the degradation of Smurf2. Overexpression of Smurf2 abolished CUEDC1 knockdown induced-EMT and the activation of TβRI/Smad signaling pathway, while siRNA Smurf2 reversed CUEDC1 overexpression-mediated regulation of EMT and TβRI/Smad signaling pathway. Additionally, CUEDC1 inhibited proliferation and promoted apoptosis of NSCLC cells. *In vivo*, CUEDC1-knockdown cells promoted metastasis and tumor growth compared with control cells. In conclusion, our findings indicate that the crucial role of CUEDC1 in NSCLC progression and provide support for its clinical investigation for therapeutic approaches.

## INTRODUCTION

Lung cancer is the leading cause of cancer mortality worldwide, and non-small cell lung cancer represents approximately 83% of lung cancer types, including lung adenocarcinoma (ADC), squamous cell carcinoma (SCC), and large cell carcinoma [1, 2]. Although different treatments, such as surgery, chemotherapy, radiotherapy and immunotherapy, have improved the prognosis of NSCLC to a certain extent, the 5-year relative survival rate is still suboptimal, largely due to metastasis [3]. Metastasis is a multi-step process that requires cancer cells to remodel the cytoskeleton and form membrane protrusions at the leading edge to switch from the epithelial state to a mesenchymal one [4, 5]. The epithelial-mesenchymal transition (EMT) is closely associated with malignant tumor migration and invasion [6]. Although abundant findings have disclosed the role of EMT on NSCLC progression, the molecular mechanisms that regulate EMT remain unclear.

The coupling of ubiquitin conjugation to endoplasmic reticulum degradation (CUE) domain was initially characterized as a mono- and poly-ubiquitin-binding motif that is required for the targeting of ubiquitinated protein to the degradation pathway [7, 8]. Notably, CUE domain proteins play critical roles in tumor progression and metastasis and EMT [9–11]. Interestingly, CUE domain-containing protein 2 (CUEDC2) is a multi-functional protein in cancer and possesses both oncogenic and tumor-suppressive properties [12, 13]. Importantly, the function of CUEDC2 appears to be different in different types of cancers [14–16]. CUEDC2 promotes breast cancer progression and endocrine resistance through the degradation of estrogen receptor-α (ERα) and the progesterone receptor (PR) [14]. In contrast, CUEDC2 inhibits glioma neurosphere formation and the tumor cell metastasis and proliferation in glioma [15]. In lung cancer, CUEDC2 also plays a tumor-suppressive role by inactivating the PI3K-Akt singling pathway [16]. However, another CUE domain protein, CUE domain-containing protein 1 (CUEDC1), has been rarely reported.

CUEDC1, 42-kDa protein assigned to chromosome 17q22, is a ubiquitously expressed protein [17, 18]. To date, little is known regarding CUEDC1, although CUEDC1 was found to be remarkably lower in a group with preeclampsia compared with the normal pregnancy group [19]. Lopes et al. reported that CUEDC1 is a functional target of ERα and is required for breast cancer cell proliferation [20]. In early-stage cervical cancer, CUEDC1 was elevated in metastasized tumors compared with non-metastasized tumors [21]. Exogenous CUEDC1 significantly promoted the tumorigenesis and malignant progression of acute lymphoid leukemia cells [22]. To date, the precise role of CUEDC1 in the metastasis and progression of NSCLC remains unknown.

Substantial data have shown that EMT is a crucial factor contributing to NSCLC invasion and metastasis [6]. Transforming growth factor-β1 (TGF-β1) is one of the strongest drivers of the EMT process and metastasis in NSCLC via the classical Smad signaling or non-canonical signaling [23–25]. In the TGF-β1 signaling pathway, TGF-β1 activates signals by binding to TGF-β type I receptor (TβRI) and subsequently activates Smad2 and Smad3 by phosphorylation. Activated Smad2 and Smad3 further interact with Smad4, and receptor-activated Smad complexes activate or repress their target gene promoters, such as Snail [26]. As previously reported, the TGF-β1 signaling pathway is regulated by Smad ubiquitin regulatory factors 2 (Smurf2) through the ubiquitination of TβRI as well as TGF-β-specific R-Smads, including Smad2 and Smad3 [27, 28]. However, CUEDC1, which plays a role in EMT, has not been reported.

In the current study, we identify a novel negative regulator of EMT and uncover a mechanism by which downregulated CUEDC1 is involved in the activation of the TβRI/Smad signaling pathway through the degradation of Smurf2, thereby promoting the metastasis of aggressive NSCLC. The purpose of this study was to explore the bio-function and potential mechanism of CUEDC1 in NSCLC progression and provide new insights into a novel potential therapeutic target for the treatment of NSCLC.

## RESULTS

### CUEDC1 acts as a metastatic and prognostic biomarker in NSCLC patients.

**Figure 1:**
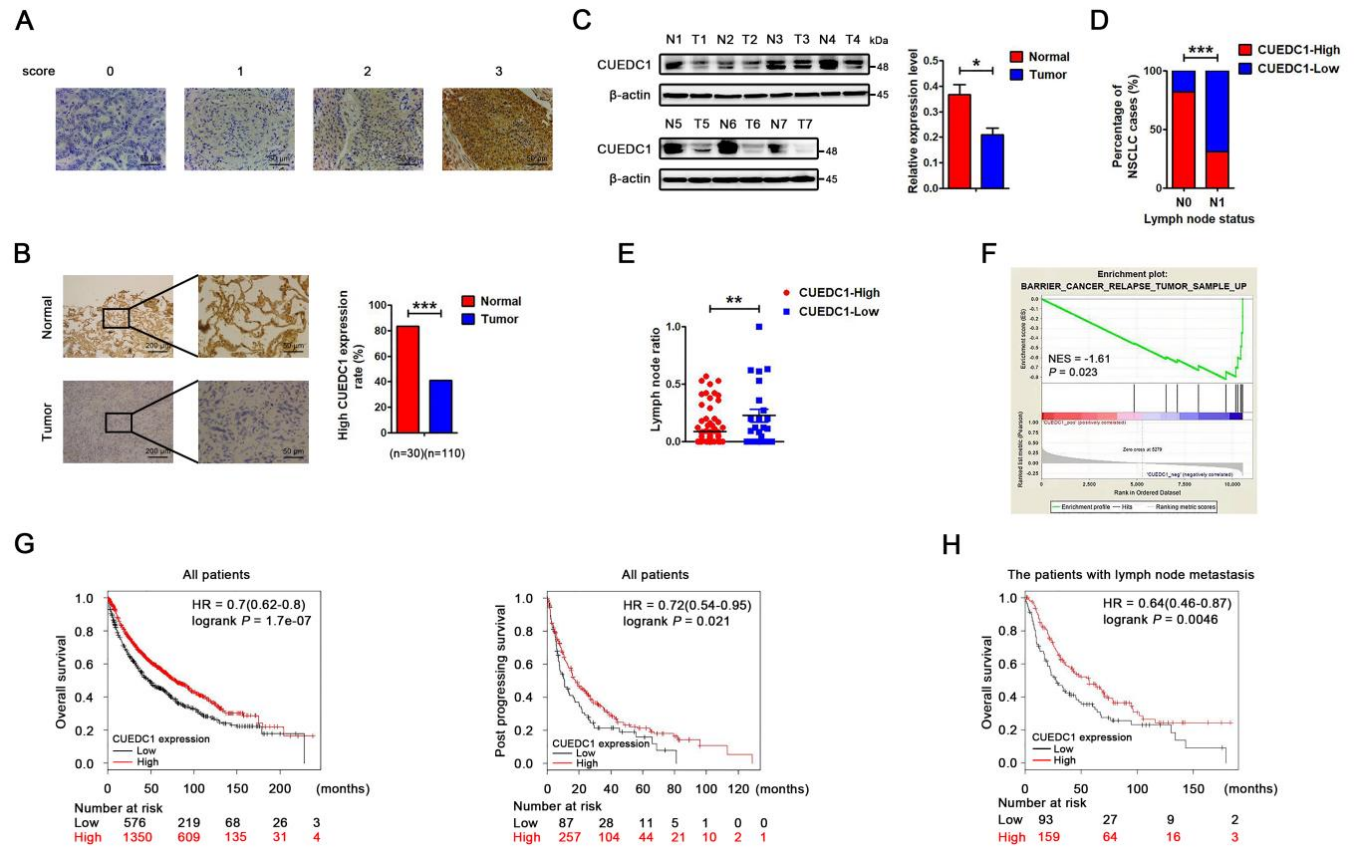
**CUEDC1 expression in lung cancer tissues.** (**A**) Immunohistochemical score of CUEDC1 expression in NSCLC and normal tissues. The staining intensity was scored with grades 0-3. (**B**) CUEDC1 expression examined by immunohistochemical analysis in 110 NSCLC patients, contained 30 pairs of NSCLC tumor tissues and their corresponding adjacent normal tissues, ***P < 0.001. (**C**) CUEDC1 expression in fresh NSCLC tumor tissues (T) and matched normal tissues (N) examined by western blotting, *P < 0.05. (**D**) Patients were classified in two groups, those with (N1) or without (N0) lymph node metastasis. IHC analysis showed that 31% of patients with lymph node metastasis had high CUEDC1 expression, whereas 82% of patients without lymph node metastasis had high CUEDC1 expression. *P* values were calculated using the χ2 test. (**E**) Analysis of the lymph node ratio (the ratio of the number of metastatic lymph nodes to the total number of examined lymph nodes) in NSCLC. *P* values were calculated using Student’s *t*-test. (**F**) The GSEA results showed a correlation between CUEDC1 levels and KEGG BARRIER CANCER RELAPSE TUMOR SAMPLE. (**G**) Kaplan-Meier survival curves of overall (left) and post progression survival (right) for high and low CUEDC1 expression levels. (**H**) Kaplan-Meier survival curves of overall survival comparing high and low CUEDC1 expression showed estimates of survival probability of lung cancer patients with lymph node metastasis using the Kaplan-Meier plotter database (219468_s_at).

To investigate the clinical significance of CUEDC1 expression in patients with NSCLC, we first examined its expression in human NSCLC tissues by IHC. CUEDC1 was clearly expressed in the cytoplasmic and nuclear compartments of tumor cells (*P* < 0.001; Figure 1A, 1B). Moreover, CUEDC1 was also significantly downregulated in NSCLC tumor tissues compared with matched surrounding tissues (*P* < 0.001; Figure 1B). Western blotting results showed that CUEDC1 expression was significantly lower in the tumor tissues than in the adjacent normal lung tissues (Figure 1C). CUEDC1 mRNA levels were detected using GEPIA in different carcinomas [29]. We first found that CUEDC1 was significantly downregulated in adrenocortical carcinoma, bladder urothelial carcinoma, colon adenocarcinoma, kidney renal clear cell carcinoma, prostate adenocarcinoma and thyroid carcinoma tissues (Supplementary Figure 1A).

Clinicopathological association analyses revealed that low CUEDC1 expression was significantly associated with lymph node metastasis (Figure 1D). Recent studies have shown that the lymph node ratio (LNR) *per se* is an independent prognostic factor for recurrence after resection of NSCLC [30]. The results showed that patients with low CUEDC1 expression level had a significantly higher LNR than patients with high CUEDC1 expression (Figure 1E). Regarding to the NSCLC pathology analysis, we showed the ratio of different pathological types and the relationship between CUEDC1 expression and different pathological types. There was no significant correlation between CUEDC1 expression and pathological type (Supplementary Figure 1B).

To elucidate the signatures of CUEDC1-correlated enriched genes, a gene set enrichment analysis (GSEA) was performed using the TCGA database in NSCLC. The GSEA results showed that CUEDC1 was negatively related with the pathway “KEGG Cancer Relapse Tumor Sample Up”, suggesting the suppressive roles of CUEDC1 in lung cancer (Figure 1F). Furthermore, the Kaplan–Meier plotter was used to assess the impact of CUEDC1 on lung cancer survival (n = 1926) [31]. The results consistently showed that patients with high CUEDC1 expression levels exhibited good overall survival (OS) and post progression survival (PPS) (Figure 1G). Elevated CUEDC1 levels may predict favorable survival for the patients with lymph node metastasis (Figure 1H). Using a stage-stratified analysis, we found that high CUEDC1 expression might be a favorable predictor for Stage I and II NSCLC (Supplementary Figure 1C). Moreover, in both male and female gender, patients with high CUEDC1 expression tended to have a longer OS than those with low CUEDC1 expression (Supplementary Figure 1D).

### CUEDC1 inhibits NSCLC cell migration and invasion

**Figure 2:**
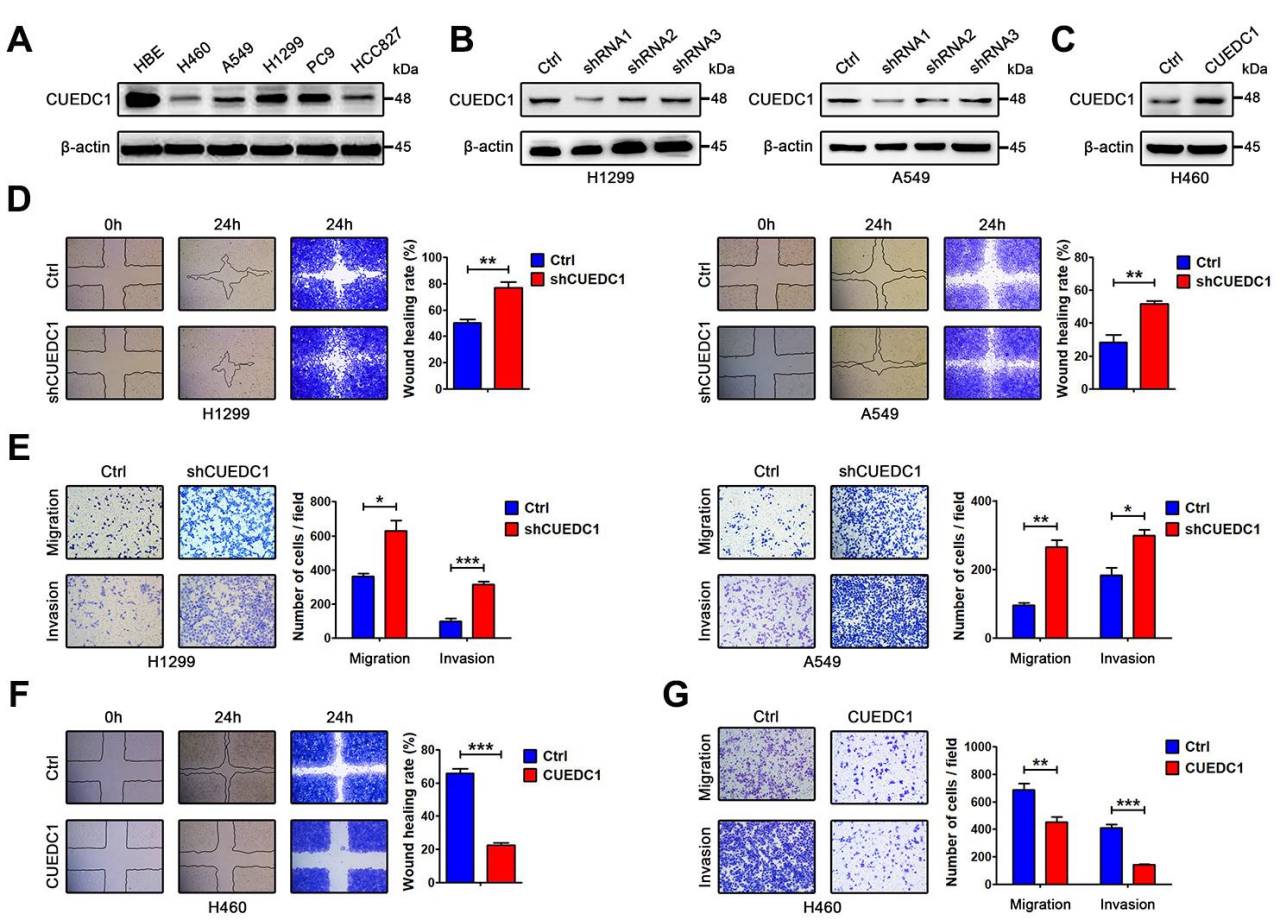
**CUEDC1 decreases the motility and invasive properties of NSCLC cells.** (**A**) CUEDC1 protein levels in different NSCLC cell lines and normal human bronchial epithelial cell line detected by western blotting. β-actin was used as an internal control. (**B**) CUEDC1 expression was confirmed by immunoblotting. CUEDC1 expression in H1299 and A549 cells was reduced markedly by shRNA1 interference. (**C**) Over-expression of CUEDC1 in H460 cells. CUEDC1 expression was determined using western blotting. (**D**) Wound-healing assays were used to investigate the migration of H1299 and A549 cells. *P* values were calculated using Student’s *t*-test. (**E**) Migration and invasion of H1299 and A549 cells (and their derivatives) were measured using transwell assays. *P* values were calculated using Student’s *t*-test. (**F**) Wound-healing assays were used to examine the migration of H460 cells. *P* values were calculated using Student’s *t*-test. (**G**) The migration and invasion of H460 cells (and their derivatives) were performed using transwell assays. The data are expressed as the mean ± SEM. *P* values were calculated using Student’s *t*-test; *P < 0.05; **P < 0.01; ***P < 0.001.

Compared with the normal human bronchial epithelial cell line HBE, low CUEDC1 expression was found in the human NSCLC cell lines (Figure 2A). To test the effect of CUEDC1 on metastasis in vitro, NCI-H1299 and A549 cells were selected as a “loss-of-function” model due to their high CUEDC1 expression, and NCI-H460 cells was selected as a “gain-of-function” model (Figure 2B, 2C). We silenced CUEDC1 expression in NCI-H1299 and A549 cells using an shRNA targeting CUEDC1. shRNA1 (CUEDC1-shRNA) was the most efficient and was therefore used in the following experiments (Figure 2B). Next, we successfully overexpressed CUEDC1 in NCI-H460 cells (Figure 2C). To assess the effect of CUEDC1 on the migration capability of NSCLC cells, a wound-healing assay was performed. The results showed that CUEDC1-shRNA significantly promoted NSCLC cell migration in both H1299 cells and A549 cells (Figure 2D). Cell proliferation can affect the results of transwell assays [32]. Cell Counting Kit-8 (CCK-8) assays were conducted to access the cell proliferation upon knockdown and overexpression of CUEDC1 (Supplementary Figure 2). We use the respective cell proliferation rates to normalized the number of migrate and invaded cells, and evaluate the ability of migration and invasion of NSCLC cells. In addition, transwell assays also showed that CUEDC1 knockdown significantly promoted lung cancer cell migration and invasion (Figure 2E). As expected, forced CUEDC1 expression markedly reduced the migratory ability and invasiveness of CUEDC1 compared with control cells in H460 cells (Figure 2F, 2G). Taken together, our results suggested that CUEDC1 could inhibit NSCLC cell migration and invasion.

### CUEDC1 negatively correlates with EMT in NSCLC tissues

**Figure 3:**
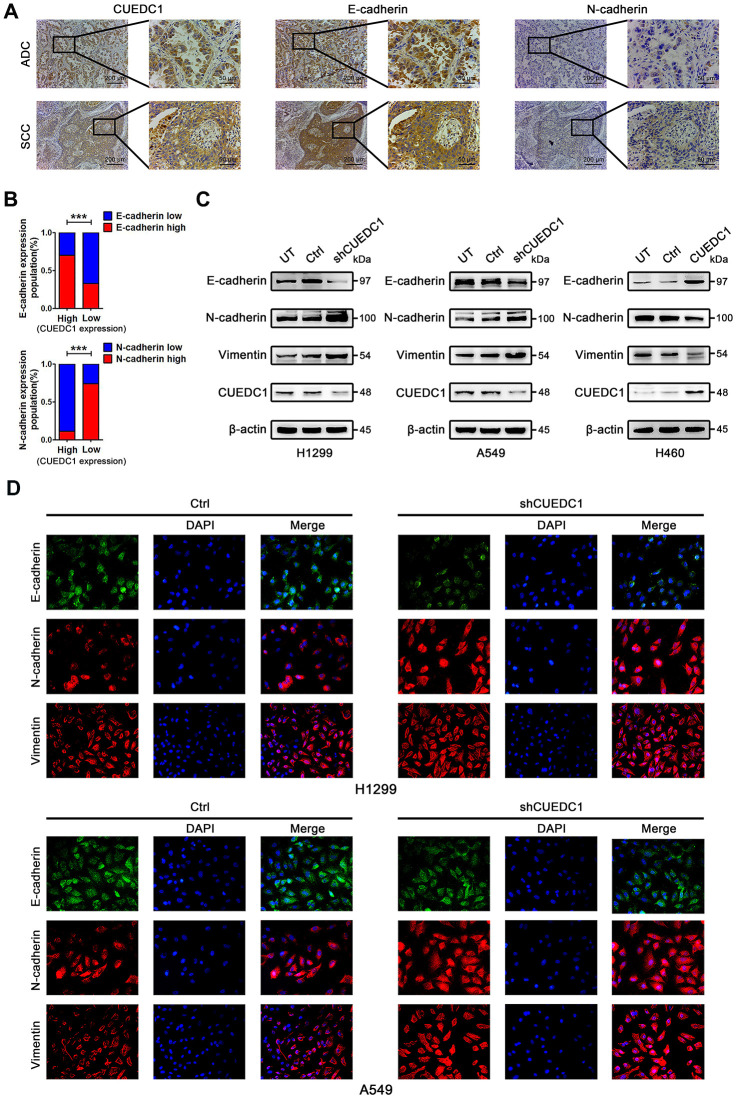
**CUEDC1 expression was negatively correlated with EMT process in NSCLC tumor tissues and cell lines.** (**A**) IHC detected the expression of CUEDC1, E-cadherin, N-cadherin in lung adenocarcinoma and lung squamous cell carcinoma tissues (magnification, ×100 and ×400). (**B**) A significant correlation between CUEDC1 expression and E-cadherin expression (upper panel), N-cadherin expression (lower panel) in NSCLC tissues. *P* values were calculated using the χ2 test. (**C**) The expression of EMT markers in CUEDC1-shRNA cells and control cells of H1299 and A549 cell lines. The expression of EMT markers in H460-CUEDC1 cells and control cells. (**D**) The protein levels and localization of EMT markers were observed by immunofluorescence in H1299 cells and A549 cells. Representative fluorescein immunocytochemical staining is depicted with E-cadherin (green), N-cadherin (red), Vimentin (red), and nuclear DAPI (blue). Experiments were performed at least three times. The data are expressed as the mean ± SEM; *P < 0.05; **P < 0.01; ***P < 0.001.

EMT is a critical step of tumor invasion and metastasis [6]. Therefore, we performed IHC assays to detect the expression of CUEDC1 and EMT markers in NSCLC tumor tissues. As expected, the tissue section staining strongly for CUEDC1 displayed high levels of E-cadherin activity but low N-cadherin expression in both ADC and SCC (Figure 3A). Next, we performed correlation analysis and found that CUEDC1 expression was positively associated with E-cadherin expression and negatively associated with N-cadherin expression (Figure 3B).

### CUEDC1 reverses the EMT program of NSCLC cells

To explore the mechanism by which CUEDC1 restrains NSCLC metastasis, we next assessed the effect of CUEDC1 on the EMT program. Knockdown of CUEDC1 repressed the expression of E-cadherin, an epithelial marker, but promoted the expression of mesenchymal markers, such as N-cadherin and Vimentin (Figure 3C). Conversely, CUEDC1 overexpression upregulated E-cadherin expression and decreased N-cadherin and Vimentin expression (Figure 3C). By immunofluorescence analysis, a reduction in E-cadherin expression and an increase in the expression of mesenchymal markers were observed in CUEDC1-depleted H1299 and A549 cells (Figure 3D). CUEDC1 expression positively correlated with E-cadherin expression in NSCLC samples by using TCGA database (Supplementary Figure 3).

### CUEDC1 inactivates the TβRI/Smad signaling pathway

**Figure 4:**
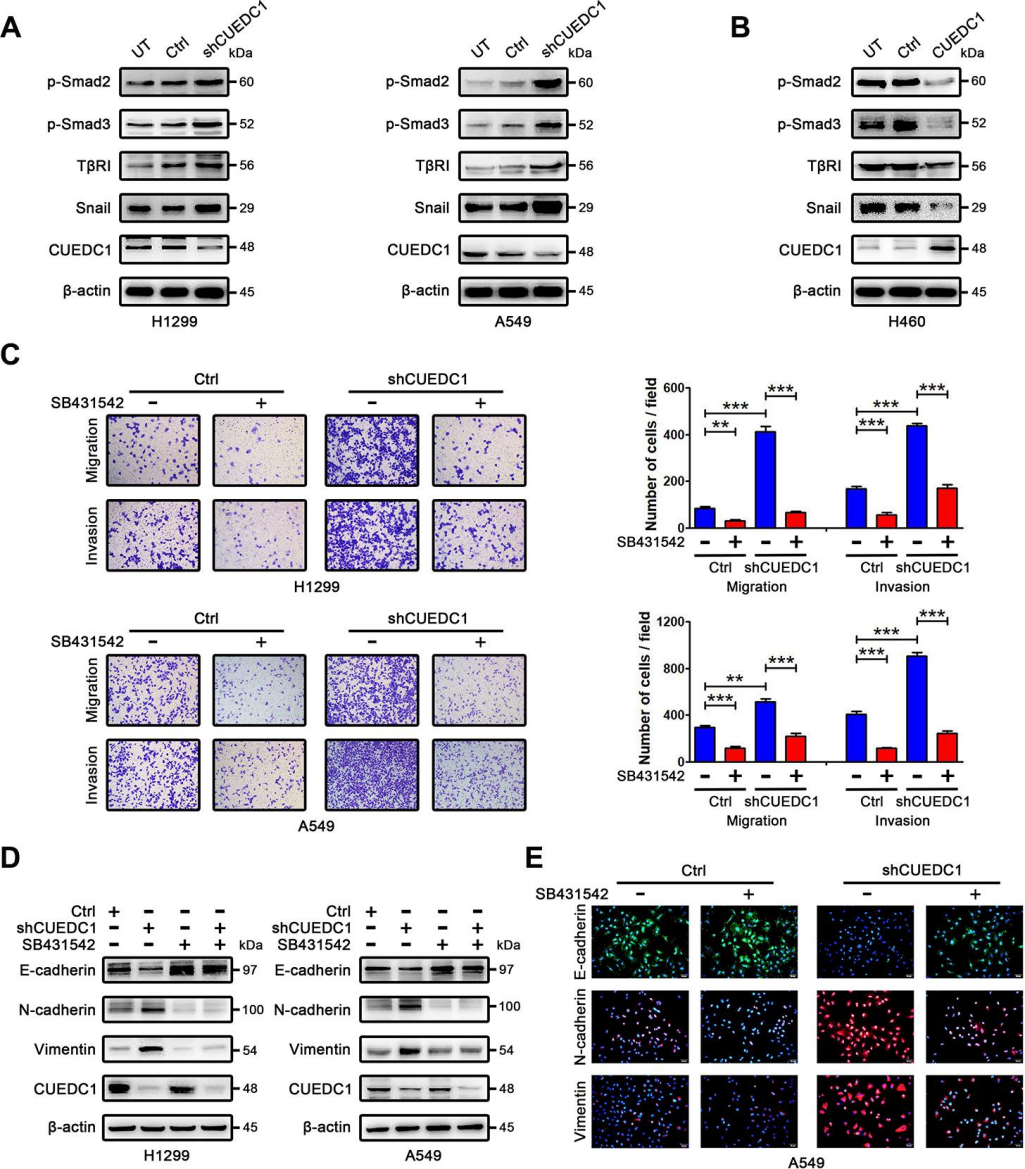
**CUEDC1 regulates TβRI/Smad signaling pathway.** (**A**, **B**) Western blotting analysis of components of the TβR I/Smad signaling pathway and Snail in NSCLC cells. (**C**) The migration and invasion ability of shRNA/CUEDC1 or negative control in H1299 and A549 cells, with or without SB431542 treatment, was detected using transwell assays. *P* values were calculated using Student’s *t*-test. (**D**) H1299 and A549 cells stably transfected with CUEDC1-shRNA or empty vector were treated with 10 μM SB431542 for 24 h, and EMT markers was determined by immunoblotting. (**E**) CUEDC1-knockdown A549 cells or negative control A549 cells were treated with or without SB431542 for 24 h. Cells were immunostained with the indicated antibodies against EMT marker proteins. Experiments were performed at least three times. The data are expressed as the mean ± SEM; *P < 0.05; **P < 0.01; ***P < 0.001.

Next, we explored a mechanism by which CUEDC1 drives the EMT progress. TGF-β pathway plays vital roles during cancer cell EMT and metastasis [26]. To determine whether CUEDC1 regulates TGF-β1 signaling pathway, enzyme-linked immunosorbent assay (ELISA) and western blotting assays were performed. As demonstrated by ELISA, TGF-β1 production was not affected by CUEDC1 in NSCLC cells (Supplementary Figure 4A, 4B). However, we found that CUEDC1 could inhibit the expression of TβRI and its downstream factors in NSCLC cells by western blotting (Figure 4A, 4B). In detail, the TβRI/Smad signaling pathway was activated when CUEDC1 was knocked down in H1299 and A549 cells (Figure 4A). CUEDC1 overexpression significantly reduced the activation of TβRI/Smad signaling pathway (Figure 4B). The expression of Snail, an EMT inducer, was also inhibited by CUEDC1 (Figure 4A, 4B).

### CUEDC1 inhibits metastasis and EMT in a TβRI-dependent manner

We subsequently evaluated whether TβRI is necessary for CUEDC1-mediated metastasis and EMT by using SB431542, a TβRI inhibitor. As illustrated in Figure 4C, SB431542 reversed the promotion of cell metastatic ability after CUEDC1 knockdown. By western blotting, we found that shRNA targeting CUEDC1 failed to induce EMT of NSCLC cells treated with SB431542 (Figure 4D). An immunofluorescent staining assay provided the same results (Figure 4E). The observations indicated that the deletion of CUEDC1 promotes metastasis and EMT in a TβRI-dependent manner.

### CUEDC1 interacts with Smurf2 and increases its expression

**Figure 5:**
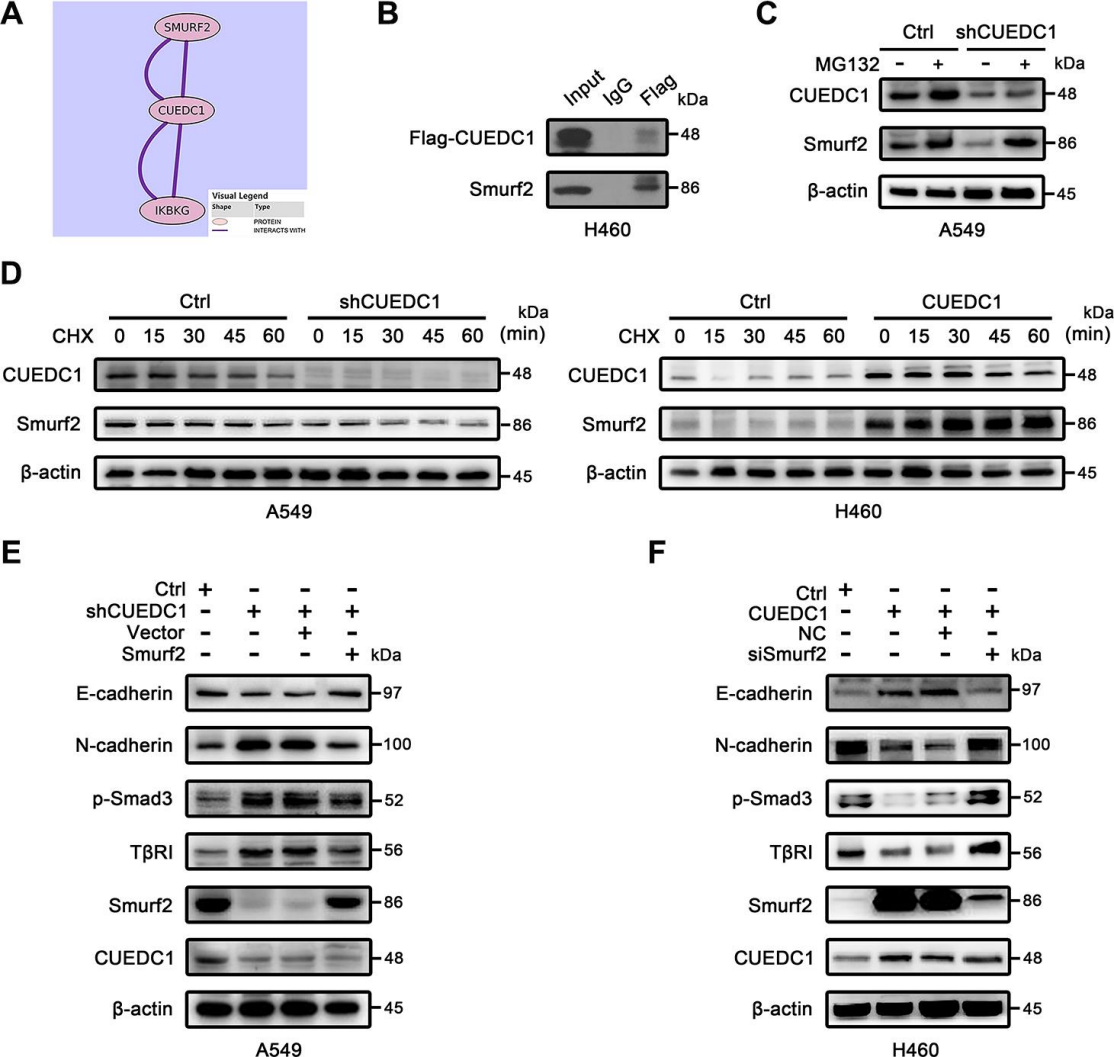
**CUEDC1 interacts with Smurf2 and inhibits TβRI/Smad signaling pathway and EMT in a Smurf2-dependent manner.** (**A**) Interaction between CUEDC1 and Smurf2 from Pathway Commons (http://www.pathwaycommons.org). (**B**) Immunoblotting analysis of lysates after immunoprecipitation from H460 cells transfected with Flag-CUEDC1. IgG was used as a negative control. (**C**) Control and CUEDC1-shRNA cells were treated with or without 10 μM MG132 for 6 h. Cell lysates were immunoblotted with the indicated antibodies. (**D**) The stability of the Smurf2 protein was measured by immunoblots in NSCLC cells treated with or without CHX. For normalization, β-actin expression was used as a control. (**E**, **F**) After Smurf2 overexpression (**E**) or knockdown (**F**), EMT markers and components of the TβR I/Smad signaling pathway were detected by western blotting.

Next, we sought to understand the underlying mechanisms how CUEDC1 inactivates the TβRI/Smad signaling pathway. From Pathway Commons (http://www.pathwaycommons.org), a “Neighborhood Map” showed the interaction between CUEDC1 and Smurf2 that was a well-known inhibitor of TβRI/Smad signaling pathway [33] (Figure 5A). Tracing to the source, a large-scale yeast two-hybrid assay mapped the Smads signaling protein–protein interactions and established a network of 755 interactions. Of these, the interaction between CUEDC1 and Smurf2 protein was discovered [34]. To further study the interaction of CUEDC1 with Smurf2, we transfected Flag-CUEDC1 in NCI-H460 cells. After the Immunoprecipitation (IP) of Flag, Smurf2 expression was detected, suggesting that CUEDC1 interacted with Smurf2 (Figure 5B).

Many CUE domain proteins regulate target proteins through the degradation pathway [7, 8]; therefore, we assessed the effect of CUEDC1 expression on the stability of the Smurf2 protein. This decreased Smurf2 stability under CUEDC1 depletion was restored by treatment with the proteasome inhibitor MG132 (Figure 5C). A cycloheximide (CHX) chase assay indicated that the half-life of Smurf2 protein was shorted after CUEDC1 knockdown in A549 cells (Figure 5D). In contrast, CUEDC1 overexpression in H460 cells prolonged the half-life of Smurf2 (Figure 5D).

Considering the impact of Smurf2 on TβRI/Smad signaling pathway [27], we next explored whether the regulation of TβRI/Smad signaling, EMT and metastasis driven by CUEDC1 depends on Smurf2. As expected, we found that the overexpression of Smurf2 abolished CUEDC1 knockdown induced-metastasis and EMT, as well as the activation of TβRI/Smad signaling pathway (Supplementary Figure 5A, 5B, Figure 5E). Consistently, knockdown of Smurf2 reversed CUEDC1 overexpression-mediated inhibition of metastasis, EMT and inactivation of TβRI/Smad signaling (Supplementary Figure 5A, 5B, Figure 5F). These results indicated that CUEDC1 negatively regulates metastasis, EMT as well as TβRI/Smad signaling pathway in a Smurf2-dependent manner in NSCLC.

### CUEDC1 knockdown promotes tumor metastasis *in vivo*

**Figure 6:**
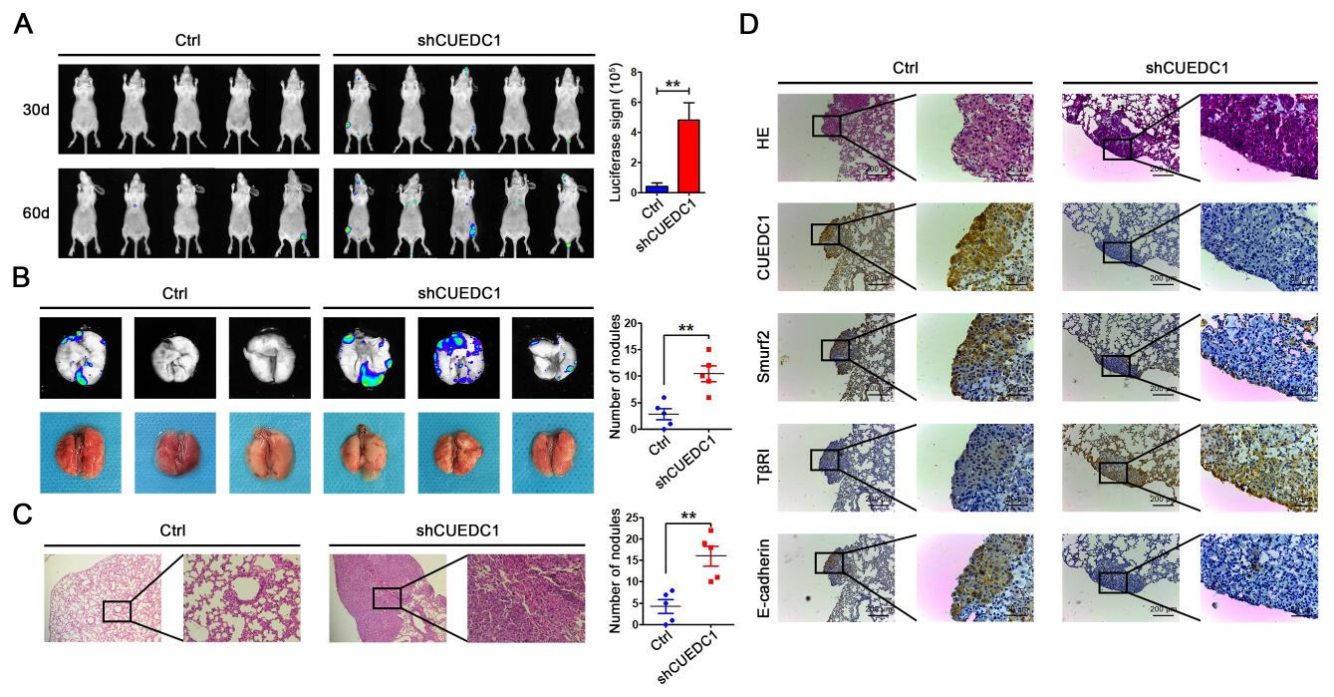
**CUEDC1 inhibited the metastasis of NSCLC *in vivo*.** (**A**) A total of 1×10^6^ of CUEDC1-knockdown or control cells were injected into tail vein of nude mice. After injection, bioluminescence images were monitored at the indicated time points. The number of metastatic sites is shown as the mean ± SEM; n=5 mice per group, *P* values were calculated using Student’s *t*-test. (**B**) The lungs were removed eight weeks later. Bioluminescence images and representative lung images of lung metastatic nodules are shown distinctly, and the numbers of metastatic nodules were measured. The data were statistically analysed by *t*-test and show the mean ± SEM. (**C**) Microscopic images of lung tissue sections stained by hematoxylin and eosin. The number of nodules on the lungs of nude mice was quantified (n=5 per group). Statistical analysis was performed using Student’s *t*-test. (**D**) CUEDC1, Smurf2, TβRI and E-cadherin expression were tested by immunohistochemical staining in lung tissues of mice (magnification, ×100 and ×400). The data are expressed as the mean ± SEM; *P < 0.05; **P < 0.01; ***P < 0.001.

To explore the effects of CUEDC1 expression level on tumor metastasis *in vivo*, lentivirus-infected A549/Ctrl cells (Ctrl) and A549/shRNA cells (shCUEDC1) were injected into mice through the tail vein (n=5, per group). Metastasis lesions were monitored by bioluminescence intensity in mice after 30 and 60 days of tail vein injection (Figure 6A). The results revealed that the tumors formed from shCUEDC1 showed higher luciferase activity than those obtained from the Ctrl in 60 days (Figure 6A). As assessed by *ex vivo* imaging, we found that the number of metastatic nodules on the surface of the lungs was increased in the shCUEDC1 group compared with the Ctrl group at 60 days post-injection (Figure 6B). Next, we performed hematoxylin and eosin staining, and the number of spontaneous lung metastatic lesions was counted in ten serial sections from each sample. A remarkable increase in the numbers metastatic foci was observed in the shCUEDC1 group compared with the Ctrl group (Figure 6C).

In addition, we found that the xenograft tissue that stained strongly for CUEDC1 also displayed high levels of Smurf2 and E-cadherin activity but expressed low levels of TβRI expression, and vice versa (Figure 6D). Consistently, in subcutaneous xenotransplanted tumor tissues, the same findings were observed (Supplementary Figure 6A). The results suggested that CUEDC1 might efficiently inhibit tumor metastasis through suppressing EMT by the regulation of the Smurf2/TβRI/Smad signaling pathway.

### CUEDC1 inhibits proliferation and promotes apoptosis of NSCLC cells

**Figure 7:**
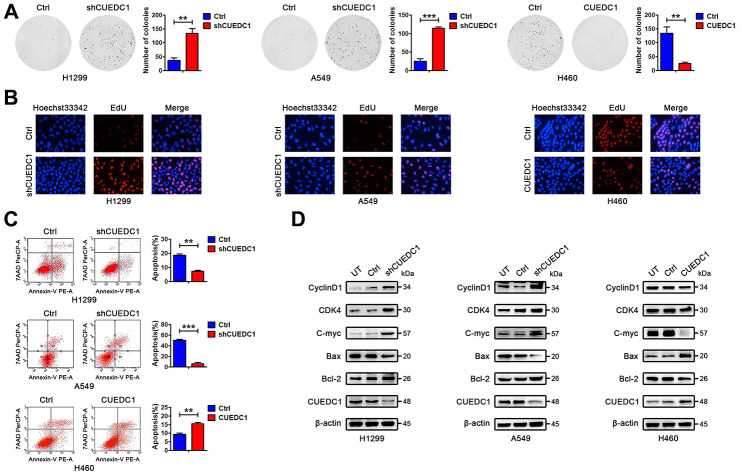
**CUEDC1 decreases cell proliferation and promotes cell apoptosis in NSCLC cells.** (**A**) Colony-forming efficiency was determined in H1299/shRNA-CUEDC1 cells, A549/shRNA-CUEDC1 cells, H460/CUEDC1 cells, and corresponding control cells (vector control). (**B**) Cell proliferation was determined by the EdU-incorporation assay in H1299/shRNA-CUEDC1 cells, A549/shRNA-CUEDC1 cells, H460/CUEDC1 cells, and corresponding control cells (vector control). (**C**) Flow cytometric analysis of apoptosis in H1299/shRNA-CUEDC1 cells, A549/shRNA-CUEDC1 cells, H460/CUEDC1 cells and negative control cells by Annexin-V and 7-AAD staining. A representative flow profile is presented (upper), and summary of % for Annexin V-positive cells is shown (below). (**D**) Western blotting detected the expression of proteins that participate in the cell cycle and apoptosis. The data are expressed as the mean ± SEM; *P < 0.05; **P < 0.01; ***P < 0.001.

To determine the effect of CUEDC1 on NSCLC cell proliferation, we conducted a CCK-8 assay. CUEDC1 knockdown increased the viability of NSCLC cells (Supplementary Figure 2). Meanwhile, CUEDC1 overexpression significantly reduced robust proliferation ability of NCI-H460 cells (Supplementary Figure 2). Additionally, we observed similar patterns in a long-term colony-formation and EdU assays, suggesting that CUEDC1 depletion promotes cell proliferation, and the expression level of cell cycle-related proteins, CyclinD1, CDK4, and C-myc, was increased (Figure 7A, 7B, 7D). Flow cytometric analysis revealed that silencing CUEDC1 decreased the percentage of apoptotic cells compared with the control cells, which was concurrent with the downregulation of the proapoptotic protein Bax and the upregulation of the anti-apoptotic protein Bcl-2 (Figure 7C, 7D). On the contrary, CUEDC1 overexpression inhibited proliferation and promoted apoptosis of NCI-H460 cells, downregulated cell cycle-related proteins and Bcl-2 expression, and upregulated Bax expression (Figure 7A–7D).

### CUEDC1 knockdown promotes tumor growth *in vivo*

**Figure 8:**
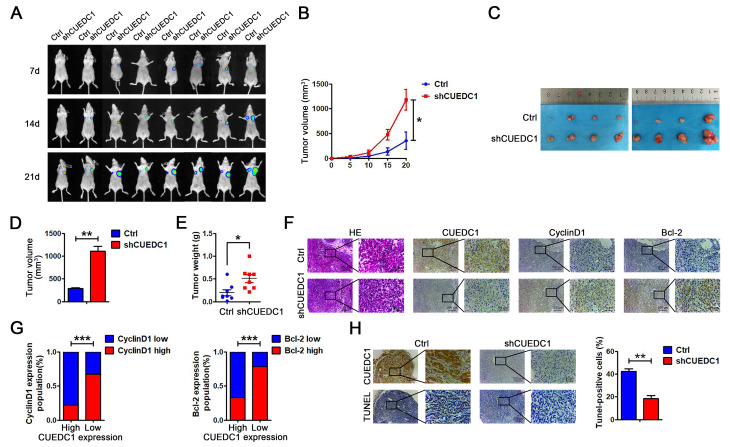
**CUEDC1 knockdown promotes cell proliferation *in vivo*.** (**A**) A total of 1 × 10^7^ of CUEDC1-knockdown A549 cells and negative control A549 cells were injected into the left and right flanks of female nude mice (n=8). After injection, bioluminescence images were monitored at the indicated time points. (**B**) Tumor growth was monitored every 5 days after implantation. The data are shown as the mean ± SEM (two-tailed Student’s *t*-test). (**C**) Images of the tumor lumps from the indicated groups at the endpoint of the experiment. (**D**, **E**) The volume (**D**) and weight (**E**) of the tumors excised are the mean ± SEM. Statistical analysis was calculated using Student’s *t*-test. (**F**) IHC analysis of CyclinD1 and Bcl-2 expression. (**G**) A significant negative correlation was observed between CUEDC1 and CyclinD1, Bcl-2 in mice tumors. Statistical analysis was performed using χ2 test. (**H**) TUNEL detection showed correlation between CUEDC1 expression and apoptosis level. The data are expressed as the mean ± SEM; *P < 0.05; **P < 0.01; ***P < 0.001.

To further investigate the tumor-suppressive role of CUEDC1 *in vivo*, we established a xenograft tumor model by subcutaneously injecting shCUEDC1 and Ctrl into nude mice. On the basis of bioluminescence imaging, we found that CUEDC1 knockdown promoted tumor growth compared with Ctrl at 7, 14, 21 days after the implantation (Figure 8A). In addition, tumor growth was monitored every 5 days after implantation. Relative to those implanted with the control cells, the mice implanted with CUEDC1-knockdown cells showed significantly increased tumor volume (Figure 8B). The mice were sacrificed at 21 days after implantation. Compared with control-treated mice, shCUEDC1-treated mice had larger tumor volumes (Figure 8C, 8D, Supplementary Figure 6B) and heavier weights (Figure 8E). IHC analyses demonstrated that CyclinD1 expression was higher in the tumor tissue obtained with shCUEDC1 than in that obtained with Ctrl (Figure 8F, 8G). Meanwhile, Bcl-2 expression was higher in shCUEDC1 tumor tissue compared with that in the Ctrl (Figure 8F, 8G). The percentage of TUNEL-positive cells obtained with the shCUEDC1 was lower than in that obtained with the Ctrl (Figure 8H). Taken together, these findings demonstrated that CUEDC1 inhibits NSCLC progression *in vivo*.

## DISCUSSION

In the present study, we found that low CUEDC1 expression was associated with the presence of lymph node metastasis and survival. We also demonstrated that CUEDC1 knockdown promoted lung cancer progression *in vitro* and *in vivo*. Our conclusion that CUEDC1 was a tumor-suppressive gene appears to conflict with the observations in breast and cervical cancer [20, 21]. In breast cancer, CUEDC1 as a cancer-promoting gene was essential for the ERα-mediated stimulation of cancer cell proliferation [20]. Such an inconsistency may be due to the differences in the tissue specificity of different type cancers. ERα has also been found to mainly localize in the nucleus of breast and cervical cancer cells [35]. In contrast to these cancer types, ERα protein expression of NSCLC is more commonly expressed in the cytoplasm compared with the nucleus [36, 37]. Most reports found that the nuclear ERα was predictive of a better prognosis, and cytoplasmic ERα was associated with a poor prognosis [37]. Through the genomic or non-genomic pathway, the cytoplasmic and nuclear ERα may have distinct functions and differentially affect prognosis [37]. Thus, we reasoned that the function of CUEDC1 may be different in different types of cancers, and the multifunctional roles of CUEDC1 in various cancers would suggest its value as a tissue-specific therapeutic target.

CUE domains were identified as ubiquitin-binding motifs and are involved in the protein degradation [38]. Similar to CUEDC1, another CUE domain-containing protein, CUEDC2, also appears to have a dual function, either as a tumor promoter or tumor suppressor [12, 13, 15, 39–42]. CUEDC2 was reported to interact with ERα and PR and promote the ubiquitination and degradation of the receptors in breast cancer [14, 42, 43]. Notably, CUEDC2 overexpression predicted a favorable clinical outcome and a longer survival time for patients with lung adenocarcinoma [16]. The similar molecular mechanism how CUEDC1 and CUEDC2 inhibit cancer progression might be they both could inactivate the canonical IκB kinase (IKK)-dependent NF-κB signaling pathway [15, 44, 45]. CUEDC2 was found interacted with IKK and repressed activation of the transcription factor NF-κB by decreasing phosphorylation and activation of IKK [15, 45]. Similarly, CUEDC1 was identified as the interactor of IKK subunit using protein microarrays, although the interactions and regulatory relationships between CUEDC1 and IKK require further validation [44]. It is interesting to explore the similar molecular mechanisms how CUEDC1 and CUEDC2 inhibit cancer progression, and these mechanisms are worthy of research in the future.

Here, we confirmed that CUEDC1 suppressed EMT in a TβRI-dependent manner. EMT plays a vital role in the early stage of tumor cell metastasis as cells lose adhesion and obtain an increased migratory and invasion ability to spread into distant tissues [6]. Multiple signaling pathways are involved in the regulation of EMT, among which the canonical TGF-β1 signaling pathway is a typical impact factor [6, 23–25]. Although aberrant CUEDC1 did not affect TGF-β1 expression, CUEDC1 had ability to inhibit TβRI expression and its downstream signaling pathway in this study. The inhibitors targeting the TβRI, such as LY-2157299 (galunisertib), EW7197 (vactosertib), SD-208 and SB431542, successfully inhibit tumors progression in preclinical models and in phase I to III clinical trials [46, 47]. Our results will help improve strategies for the selection of NSCLC patients who may particularly benefit from agents that selectively target TβRI.

In this study, we found that CUEDC1 interacts with Smurf2 and positively regulates its expression in NSCLC. Previous studies reported that the duration and stability of TGF-β1 signaling pathway is tightly inhibited by Smurf2, the C2-WW-HECT E3 ligases [27]. The WW domains mediates the interactions between HECT type E3 ubiquitin ligase and proline-containing PPxY motifs of its substrates (i.e., Smad2/3) [48]. It was previously demonstrated that SMAD undergoes SMURF2-mediated multi-monoubiquitination that blocks SMAD nuclear translocation, thus activity, instead of polyubiquitination and degradation [49]. This finding may explain why TβRI, p-Smad2 and p-Smad3 protein levels were reduced by CUEDC1 in this study.

We also validated that CUEDC1 promoted apoptosis and inhibited the proliferation of NSCLC cells. Bax acts as a proapoptotic gene in intrinsic apoptotic pathway. In contrast, antiapoptotic proteins (e.g., Bcl-2) inhibit proapoptotic factors to preserve outer mitochondrial membrane integrity. C-myc is the first proto-oncogene discovered and is known to participate in many cellular functions, including driving the G0- G1transit [50]. CyclinD1 and CDK4 serve as cell cycle regulatory switch in actively proliferating cells [50]. Although we discovered that CUEDC1 inhibited Bcl-2, C-myc, CyclinD1 and CDK4 expression and facilitated Bax expression, we did not fully elucidate the detailed mechanism by which CUEDC1 promotes tumor growth. We will explore the mechanism further in future studies.

**Figure 9:**
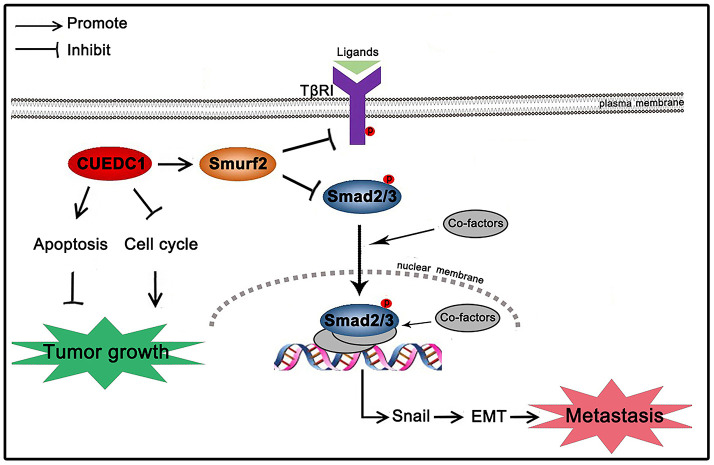
**Schematic mechanism underlying CUEDC1-suppressed NSCLC progression.** CUEDC1 interacts and upregulates Smurf2 expression; then, Smurf2 inactivates the TβRI/Smad signaling pathway, thereby reversing EMT and reducing the ability for tumor metastasis, inhibiting cell cycle progression, and promoting cell apoptosis to suppress tumor growth.

In summary, the present study showed a working model for how CUEDC1 inhibits NSCLC tumor growth and metastasis (Figure 9). CUEDC1 interacts with Smurf2 and upregulates Smurf2 expression, thereby inactivating the TβRI/Smad signaling pathway. The inactivated TβRI/Smad signaling pathway impaired EMT and metastasis. In addition, CUEDC1 inhibits proliferation and promotes apoptosis of NSCLC cells, blocking tumor growth (Figure 9). This study may highlight a new therapeutic opportunity for treating advanced NSCLC by targeting CUEDC1.

## MATERIALS AND METHODS

### Cell culture and tissue specimens

Human NSCLC cell lines were cultured in RPMI-1640 and DMEM with 10% fetal calf serum, and 1% penicillin/streptomycin. HCC827, H1299, A549, H460, PC9 were maintained in a humidified atmosphere at 37° C with 5% CO_2._ Cell lines were corroborated periodically using short-tandem repeat (STR) fingerprinting assay. Clinical samples were acquired from 110 NSCLC patients who were treated by surgical removal of the cancer at Harbin Medical University Cancer Hospital from January 2010 to August 2016. The details of the patients' population in the study were systematically presented in Supplementary Table 1. Tissues were fixed with formalin and embedded in paraffin. 7 pairs of fresh lung tissues (paired NSCLC tumor samples and matched adjacent normal tissue samples) were resected from 7 NSCLC patients who underwent surgical lung resection between August 2017 to August 2018. Normal lung tissue samples were taken from areas a standard distance (3 cm) from resected NSCLC patients [51]. Ethical clearance and approval were obtained from the Ethics Review Committee at Harbin Medical University.

### Immunohistochemistry staining

IHC was performed as previously described [52]. Antibodies against CUEDC1 (GTX85378, GeneTex, 1:100), E-cadherin (20874-1-AP, Proteintech, 1:5000), N-cadherin (22018-1-AP, Proteintech, 1:1000), Smurf2 (A10592, ABclonal, 1:100), TβRI (A0708, ABclonal, 1:100), CyclinD1 (26939-1-AP, Proteintech, 1:100) and Bcl-2 (26593-1-AP, Proteintech, 1:100) were used for IHC.

### Chemicals and lentivirus infection

The overexpression sequence of CUEDC1 was devised, and lentiviruses carrying small hairpin RNA (shRNA) were packaged by HANBIO Company (Shanghai, China). A total of 3×10^5^ cells were seeded into six-well plates and maintained 37° C overnight. The next day, the cells were washed with PBS, and 1 ml of medium was added to each plate. Then, 6 μl of lentivirus and 2.5 μl of polybrene were added directly to the medium. After 16 hours, the medium was replaced. The design of the knockdown CUEDC1 sequence and the packaging of lentiviruses were completed by GeneChem Company (Shanghai, China). The human CUEDC1 shRNA-specific target sequences used are as follows: shRNA1 (5’-GGAUUACGACAUCGAATT-3’), shRNA2 (5’-GG ACCUGAUAGCUCGGAUTT-3’); and shRNA3 (5’-GCAACCUUCCGGAUGACU UTT-3’). The vector sequence was used as a control. Cells were planted in six-well plates the day before infection. After washing with PBS, 2.5 ml of medium were added to each plate. Then, 8 μl of lentivirus and 100 μl of HitransG P were added directly to the medium. After 4 hours, 1 ml of medium was added; 16 hours later, the medium was replaced. At 48 hours after transfection, puromycin was added to the medium for one week to select stably infected cells. Smurf2 was silenced with siRNA (RiboBio, Guangzhou, China), according to the manufacturer’s instructions; the target sequences were as follows: siRNA (5’-GCAGACCTCTTAGCTGCTTTG-3’). The corresponding negative control was purchased from RiboBio Co., Ltd. In this study, 10 μM SB431542 (HY-10431, MCE), 50 μg/ml CHX (HY-12320, MCE) and 10 μM MG132 (HY-13259, MCE) were used.

### Western blotting analysis

Cells were lysed using RIPA lysis buffer containing protease inhibitors to obtain protein. A BCA Protein Assay Kit was used to determine the protein concentrations. Proteins were separated by 10% SDS-polyacrylamide gel electrophoresis and transferred to PVDF membranes. Subsequently, the membranes were blocked with 5% BSA blocking reagent for 1 hour at RT and incubated with primary antibodies overnight at 4° C. The next day, the membranes were washed and incubated for 1 hour at RT with secondary antibodies. Finally, the proteins were analysed using the ECL Plus kit. The antibodies used included the following: CUEDC1 (ab58696, Abcam, 1:1000); Flag antibody (#14793, Cell Signaling Technology, 1:50); E-cadherin (ab40772, Abcam, 1:1000); N-cadherin (ab18203, Abcam, 1:1000); Vimentin (ab92547, Abcam, 1:1000); Smurf2 (ab53316, Abcam, 1:1000); TβRI (A0708, ABclonal, 1:1000); Snail (#3879, Cell Signaling Technology, 1:1000); p-Smad2 (Ser465/467, #3108, Cell Signaling Technology, 1:1000); p-Smad3 (Ser423/425, #9520, Cell Signaling Technology, 1:1000); CyclinD1 (26939-1-AP, Proteintech, 1:1000); CDK4 (#12790, Cell Signaling Technology, 1:1000); C-myc (A1309, ABclonal, 1:1000); Bax (A15646, ABclonal, 1:1000); Bcl-2 (26593-1-AP, Proteintech, 1:1000); and β-actin (TA-09, ZSGB-BIO, 1:1500).

### Tumor cell migration and invasion assays

The cells were allowed to reach 100% confluence in six-well plates. Then, a 10-μl pipette tip was used to create a wound, and the cells were washed with PBS two times. The speed of wound healing was observed and imaged using a microscope, and the rate of closure was estimated. For the migration assay, 3×10^4^ cells were added to the upper chamber, and the cells were allowed to migrate to the lower chamber for 24 hours. The cells were fixed with cold 4% paraformaldehyde (PFA) for 30 min and stained with 0.1% crystal violet for 30 min. Five random fields of each membrane were selected; images were captured, and the number of migrated cells was counted. For the invasion assay, 3×10^4^ cells were added to the upper chamber, which was pre-coated with Matrigel. After 48 hours of invasion, the chambers were treated as above. Normalized invasion cell or migration cell number = actual invasion cell or migration cell number/cell growth rate.

### Immunofluorescence

Immunofluorescence assays were performed as previously described [52]. After blocking with 5% BSA, the cells were incubated with the primary antibodies E-cadherin (ab40772, Abcam, 1:500), N-cadherin (ab18203, Abcam, 1:200) and Vimentin (ab92547, Abcam, 1:250) at 4° C overnight. The next day, the cells were washed and incubated with secondary antibodies for 1 hour at RT. Finally, the nucleus was stained with DAPI. All images were acquired using an inverted fluorescence microscope.

### Enzyme-linked immunosorbent assay

The TGF-β1 concentrations in the CMs were detected using a human TGF-β1 ELISA kit (RK00055, ABclonal). The measurements were performed in accordance with the instructions provided by the manufacturer, and the OD values were detected using a microplate reader at 450 nm.

### Tumor xenograft model in nude mice

Female BALB/c athymic nude mice (4–5 weeks old) were purchased from Beijing Vital River Laboratory Animal Technology Co., Ltd and raised in pathogen-free conditions at the Animal Center of the Second Affiliated Hospital of Harbin Medical University. A549/Ctrl cells (Ctrl) and A549/shRNA cells (shCUEDC1) were separately injected into the left and right flanks of mice to monitor tumor growth (n=8). For metastasis analysis, CUEDC1-depleted A549 cells or control cells were injected into the tail vein of mice (n=5, per group). The mice were intraperitoneally injected with D-luciferin, and bioluminescence images were obtained to monitor the primary tumor growth and the occurrence of metastasis. Xenografted tumor size was monitored every 5 days (volume = length × width^2^ × 0.52). The mice were euthanized after four weeks or eight weeks to check primary tumor growth and the presence of lung metastasis. In metastasis experiments, the mice were randomly assigned to the experimental groups. After all mice were sacrificed, the tumor tissue was removed, soaked in formalin and embedded in paraffin for IHC analysis. All animal experiments were performed in conformity to the NIH Guide for the Care and Use of Laboratory Animals and the Institutional Animal Care and Use Committee (IACUC) of Harbin Medical University in China.

### Immunoprecipitation

Immunoprecipitation analysis was accomplished as described [52]. Briefly, cells with overexpressed Flag-CUEDC1 were fully lytic with IP lysis buffer (Thermo Fisher Scientific, MA, USA) and immunoprecipitated with a Flag antibody. The lysates and precipitates were analysed by western blotting to detect CUEDC1, Smurf2 and β-actin protein expression.

### Cell proliferation assays

A total of 5×10^3^ cells were seeded into 96-well plates and cultured at 37° C. For cell viability analysis, 10 μl of Cell Counting Kit-8 was mixed with 90 μl of medium, and the mixture was added to each well and maintained for 1 hour at 37° C; then, absorbance was measured at 450 nm. Three independent experiments were performed. Alternatively, the EdU-incorporation assay was used to detect cell proliferation. A total of 5×10^3^ cells were seeded in 96-well plates; the next day, 50 μM EdU (Ribobio, Guangzhou, China) was added and maintained at 37° C for 4 hours. The cells were fixed in 4% formaldehyde for 30 min and subsequently permeabilization in 0.5% Triton X-100 for 10 min at RT. After that, the cells were washed with PBS and incubated with 1XApollo® reaction cocktail (100 μl per well) for 30 min. Nuclear DNA was stained with Hoechst 33342 (5 μg/ml) for 30 min. Images were observed under an inverted fluorescence microscope.

### Apoptosis analysis

For apoptotic analysis, the cells were stained using Annexin V-PE/7-AAD Apoptosis Kit (559763; BD Biosciences), and apoptosis was evaluated by flow cytometry in accordance with the manufacturer’s protocol. Apoptotic cells in the xenograft tumors were identified by terminal deoxynucleotidyl transferase-mediated dUTP nick end-labelling (TUNEL) staining using the In Situ Cell Death Detection Kit (11684817910, Roche). The average number of stained cells in three images from each treatment group was calculated.

### Statistical methods

All data are representative of at least three independent experiments. The data are presented as the mean ± the standard error of the mean (SEM) as indicated. Statistical analysis was conducted using Student’s t-test and χ^2^ tests, and *P* < 0.05 was considered significant. All statistical analyses were performed using GraphPad Prism 5 software and SPSS 22.0 software. Survival curves were plotted using the Kaplan–Meier method and compared using the log-rank test.

## Supplementary Material

Supplementary Figures

Supplementary Table 1
