# Supplementary material for: CUEDC1 inhibits epithelial-mesenchymal transition via the TβRI/Smad signaling pathway and suppresses tumor progression in non-small cell lung cancer
Source: Aging (Albany NY). 2020 Oct 25;12(20):20047–68. doi: 10.18632/aging.103329 (PMC7655170; doi:10.18632/aging.103329)
Supplement: Supplementary Figures [file aging-12-103329-s001..pdf]

SUPPLEMENTARY FIGURES

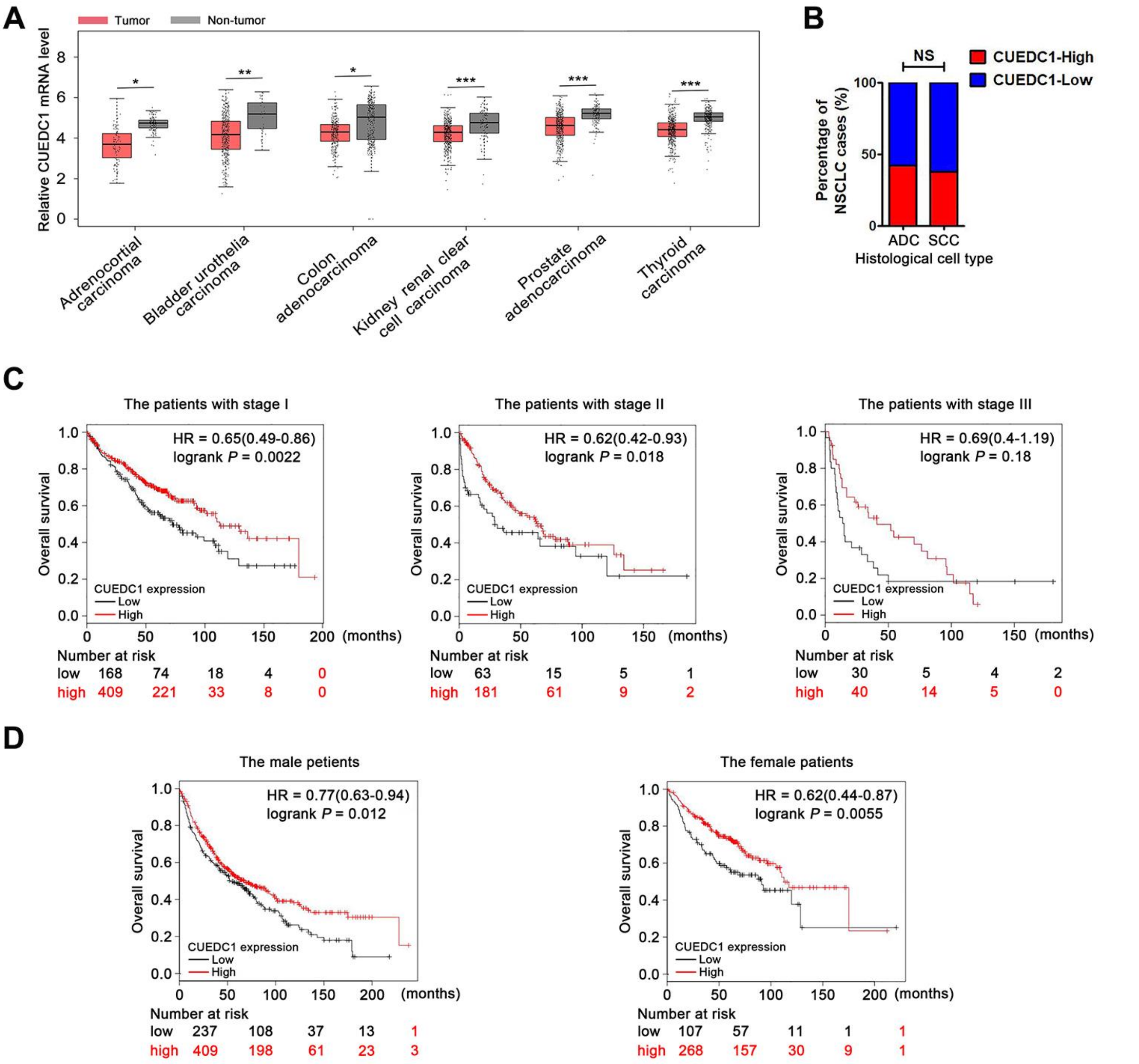

Supplementary Figure 1. CUEDC1 expression in tumor tissues is lower than that in normal tissues and predicts good patient prognosis. (**A**) GEPIA show CUEDC1 expression levels in different carcinomas. (**B**) IHC analysis showed that 42% of patients with adenocarcinoma had high CUEDC1 expression, whereas 38% of patients with Squamous cell carcinoma had high CUEDC1 expression. *P* values were calculated using the χ^2 test. (**C**, **D**) Correlation between CUEDC1 expression and clinical outcomes. A Kaplan–Meier plot analysis showed overall survival of lung cancer patients in different clinical stages (**C**) and lung cancer patients in different gender (**D**) depending on CUEDC1 expression. *P* values were calculated using a log-rank test.

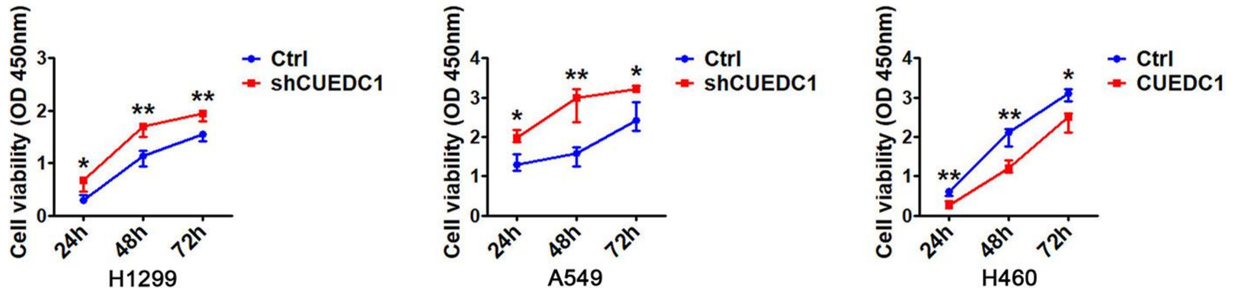

Supplementary Figure 2. CUEDC1 inhibits NSCLC cell proliferation. CCK-8 assays were performed in H1299 and A549 cells silenced for CUEDC1 and performed in H460 cells overexpressing CUEDC1. *P* values were calculated using Student's *t*-test. The data are expressed as the mean ± SEM; **P* < 0.05; ***P* < 0.01; ****P* < 0.001.

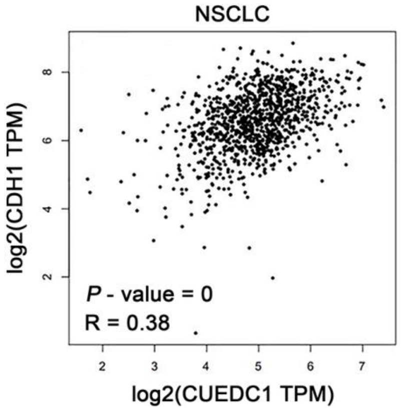

Supplementary Figure 3. CUEDC1 expression positively correlated with E-cadherin. The correlation between expression of CUEDC1 and E-cadherin in NSCLC samples using TCGA database.

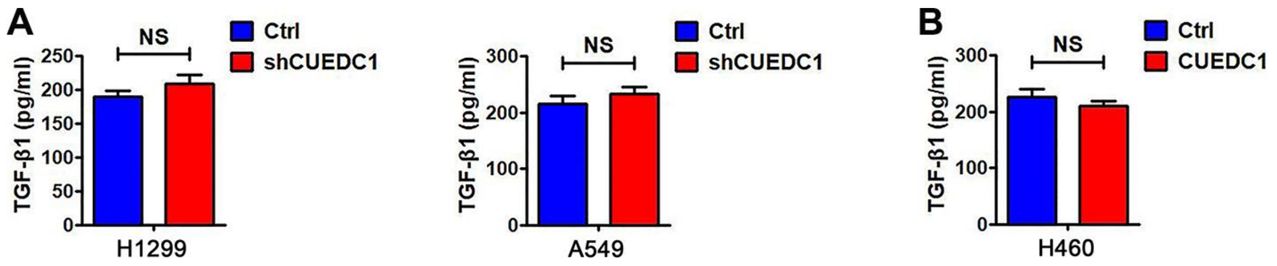

Supplementary Figure 4. CUEDC1 had no effect on the secretion of TGF-β1 in NSCLC cells. (**A**, **B**) Levels of TGF-β1 in culture medium of H1299/shRNA-CUEDC1 cells, A549/shRNA-CUEDC1 cells (**A**), and H460/CUEDC1 cells (**B**), and corresponding control cells (vector control). *P* values were calculated using Student's *t*-test; **P* < 0.05; ***P* < 0.01; ****P* < 0.001.

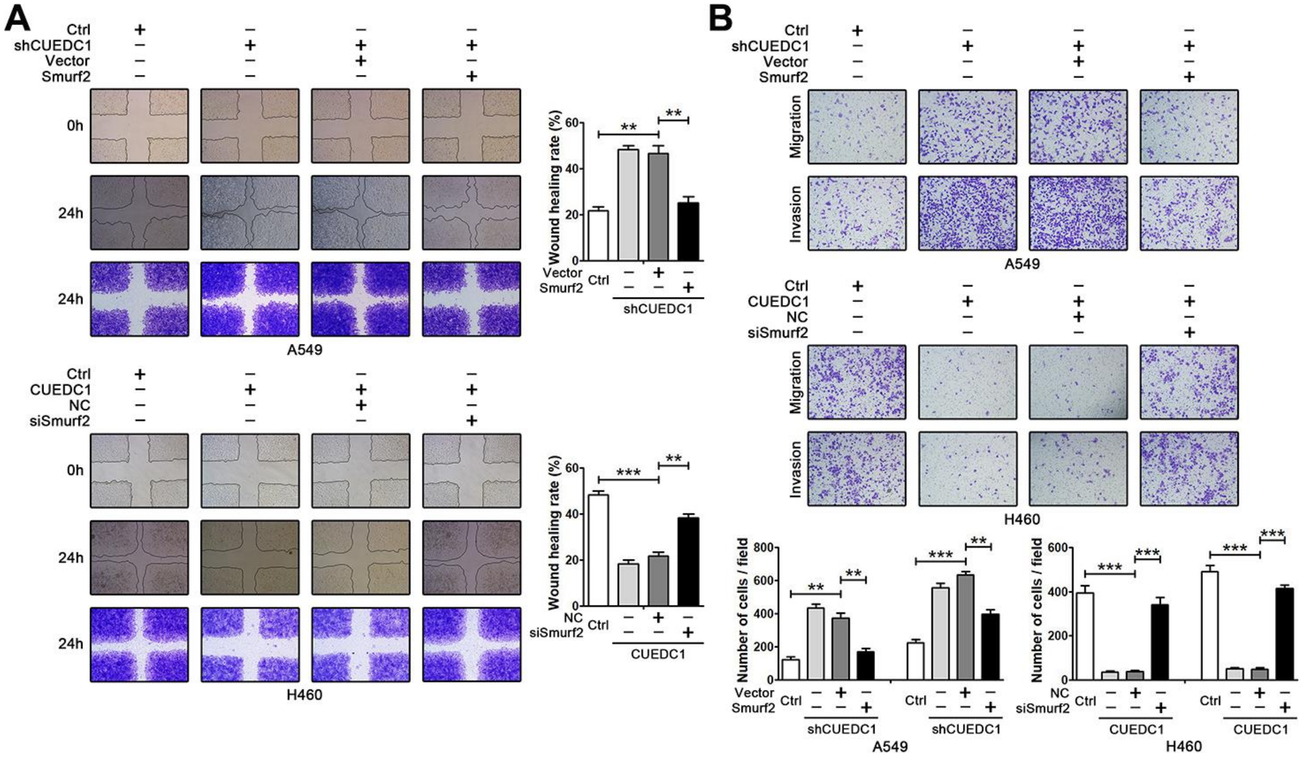

Supplementary Figure 5. CUEDC1 inhibits metastasis in a Smurf2-dependent manner. (**A**, **B**) After Smurf2 overexpression or knockdown, migration and invasion of A549 and H460 cells were measured using wound-healing assays (**A**) or transwell assays (**B**). The data are expressed as the mean ± SEM. *P* values were calculated using Student's *t*-test; *$P < 0.05$; **$P < 0.01$; ***$P < 0.001$.

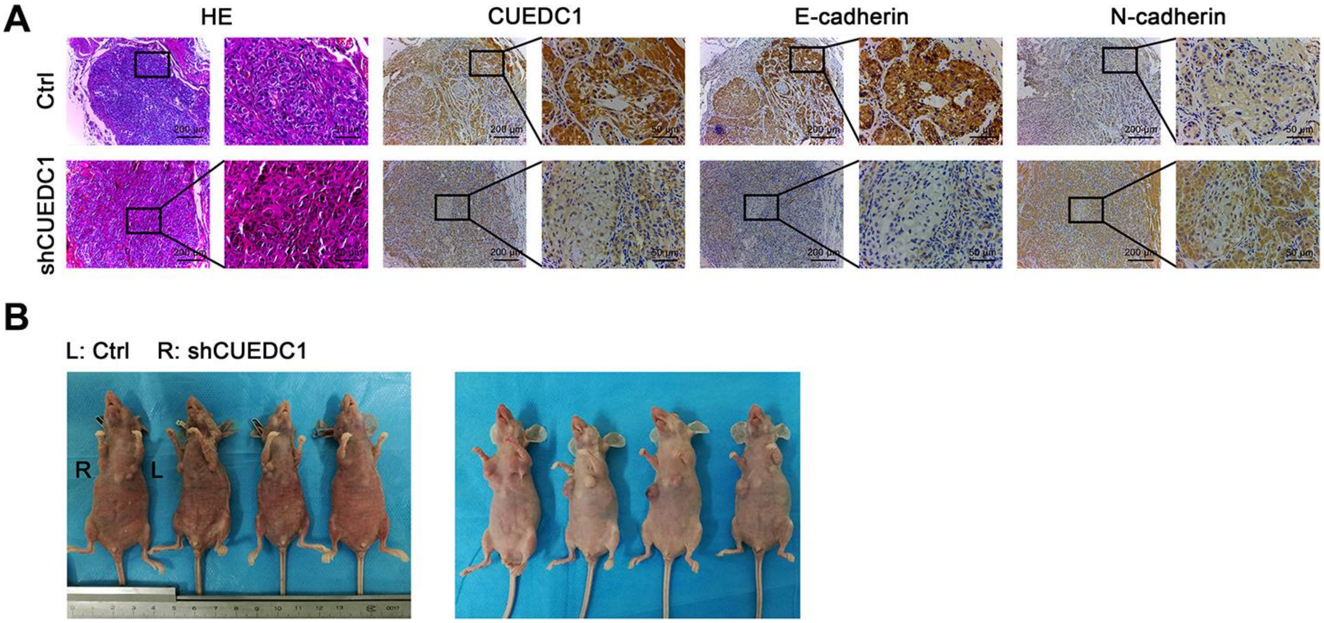

Supplementary Figure 6. Deletion of CUEDC1 promotes the growth of tumors and the EMT process. (**A**) The expression of EMT markers was determined by immunohistochemical staining in xenograft tumor of mice (magnification, ×100 and ×400). (**B**) CUEDC1 downregulation results in increased cell proliferation of NSCLC cells *in vivo*. Images of the tumor on mice.
