# Supplementary material for: CUEDC1 inhibits epithelial-mesenchymal transition via the TβRI/Smad signaling pathway and suppresses tumor progression in non-small cell lung cancer
Source: Aging (Albany NY). 2020 Oct 25;12(20):20047–68. doi: 10.18632/aging.103329 (PMC7655170; doi:10.18632/aging.103329)
Supplement: Supplementary Table 1 [file aging-12-103329-s002..pdf]

SUPPLEMENTARY TABLE

Supplementary Table 1. The basic information of 110 patients with NSCLC for CUEDC1 immunohistochemical staining analysis.

		Cases (n)	Percentage (%)
Histological cell type	Adenocarcinoma	77	70
	Squamous cell carcinoma	33	30
Gender	Male	73	66
	Female	37	34
Age (years)	<60	72	65
	≥60	38	35
Differentiation	Well	22	20
	Moderate	45	41
	Poor	43	39
pTNM Stage	I	46	42
	II	37	34
	III	27	24
	IV	0	0
pT classification	T1	37	34
	T2	64	58
	T3/4	9	8
N classification	N0	61	55
	N1	14	13
	N2	35	32
M classification	M0	110	100
	M1	0	0

Abbreviations: NSCLC = non-small cell lung cancer; pTNM stage = tumor, node, metastasis (pathological stage); pT = pathological T stage; n = number of patients.
